# Seasonal Correlations Between UV Exposure and Search Trends for Mites and Rosacea in Shanghai

**DOI:** 10.1111/jocd.70184

**Published:** 2025-04-21

**Authors:** Sitong Li, Jiacheng Lin, Jiaqi Li, Xiaohui Mo, Qiang Ju

**Affiliations:** ^1^ Department of Dermatology Renji Hospital, Shanghai Jiao Tong University School of Medicine Shanghai People's Republic of China

**Keywords:** Baidu index, demodex mites, erythemal UV dose, mite, rosacea, ultraviolet radiation

To the Editor,

1

Online search data, such as Google Trends and Baidu Index, offer valuable insights into skin conditions, including seasonal trends in itch and the links with air pollution [1, 2]. Mites, microscopic arthropods in the subclass Acari, are associated with various skin diseases, with demodex mites linked to rosacea [3]. Given the influence of UV radiation on skin conditions, this study examines the relationship between Baidu search queries for mites, rosacea, and UV radiation levels in Shanghai, China. By analyzing the correlations between these search trends and UV exposure, we aim to uncover seasonal patterns and explore how UV radiation influences public interest in these skin concerns.

We extracted Baidu Index data on mites and rosacea from https://index.baidu.com/v2/index.html, and erythemal UV dose (UVD) data, as a measure of UV radiation levels, from the Tropospheric Emission Monitoring Internet Service (TEMIS) at https://www.temis.nl/uvradiation/UVdose.php. The dataset spans from January 1, 2015, to December 31, 2019.

Seasonal decomposition analysis with a 365‐day cycle reveals strong seasonal patterns in both mite searches and UVD data. Rosacea searches trends exhibit weaker seasonality, consistent with previously reported rosacea consultation pattern [4]. The steady annual increase in rosacea‐related searches reflects growing public awareness and concern (Figure 1).

**FIGURE 1:**
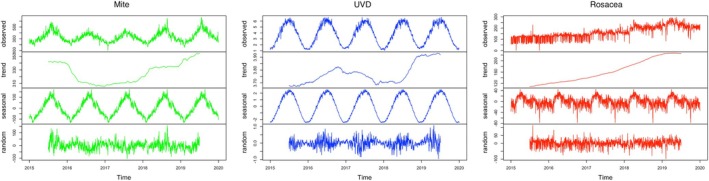
Additive Seasonal Decomposition of Mite, Rosacea, and Erythemal UV Dose Data. The sure presents the additive seasonal decomposition of mite, rosacea, and erythemal ultraviolet dose (UVD) data from January 1, 2015, to December 31, 2019. The green line represents mite query data, the red line represents rosacea query data, and the blue line represents UVD data from TEMIS. Each dataset is decomposed into four components: Observed data, estimated trend, seasonal pattern, and residuals. The Baidu Index for mites and erythemal UV dose display distinct seasonal patterns, whereas the Baidu Index for rosacea shows a consistent annual increase.

This study used Pearson correlation analysis to assess the linear relationship between two variables; however, this method does not reveal causal relationships. Therefore, the Granger causality test was applied to explore causal relationships in the time series data. Pearson correlation analysis of seasonal components revealed a strong positive correlation between UVD and mite search queries (*r* = 0.787, *p* < 0.05), indicating that both may be influenced by similar seasonal factors. A moderate positive correlation was observed between UVD and rosacea searches (*r* = 0.295, *p* < 0.05), suggesting an association between UV exposure and rosacea. However, the correlation between mite and rosacea queries was very weak (*r* = 0.052, *p* < 0.05), likely due to distinct influencing factors, nonlinear relationships, or potential lag effects.

Granger causality tests showed that diff_UVD significantly Granger‐causes diff_mite (*F* [28, 3480] = 2.5609, *p* = 1.246 × 10^−5^), indicating that UV exposure changes predict fluctuations in public interest in mites. Additionally, diff_mite significantly Granger‐causes rosacea (*F* [14, 3564] = 5.2993, *p* = 4.358 × 10^−10^), demonstrating a strong temporal association between interest in mites and subsequent interest in rosacea. Although diff_UVD also Granger‐causes rosacea (*F* [30, 3468] = 1.487, *p* = 0.04291), the *p*‐value is close to 0.05, suggesting a weaker predictive relationship, likely due to the mediator effects of mites, other environmental factors, or individual differences in rosacea patients' UV responses. Instantaneous causality was found only between mite and rosacea queries (*p* = 5.255 × 10^−5^), with no significant instantaneous effects observed between other pairs.

UV radiation impacts skin conditions [5], with studies showing that demodex mite infestations tend to worsen in summer [6]. One possible explanation is that increased UV exposure alters the skin microbiome or immune environment, indirectly affecting mite proliferation. A clinical study has shown that patients undergoing phototherapy have a significantly higher prevalence of Demodex folliculorum infestation compared to healthy controls, particularly those receiving PUVA treatment, suggesting that phototherapy may facilitate Demodex proliferation [7]. We hypothesize that UV exposure may exacerbate mite‐related pathogenesis, leading to increased public interest in mites and, subsequently, rosacea. A key biological factor may be the absence of UV‐protection genes in Demodex mites, making them particularly vulnerable to increased UV radiation [8]. This vulnerability might drive them to migrate deeper into hair follicles, triggering inflammatory responses in susceptible individuals. Moreover, UV exposure can impact mite embryonic development, influencing their population dynamics [9]. Given UV exposure's potential to worsen mite‐related skin conditions, proactive screening, sun protection, and mite management may be beneficial. Individuals prone to mite infestations, such as those with papulopustular rosacea (PPR), should adopt stricter UV protection measures.

The Baidu Index is a valuable tool for analyzing public interest in health issues. However, varying public awareness about different types of mites results in low search volumes for specific mites (e.g., Demodex mites). Therefore, this study used the broader keyword “mites”, providing a more comprehensive reflection of public interest in mite‐related issues. Despite its utility, public search trends as a proxy for disease prevalence have inherent limitations. Search volumes are influenced by media coverage, public awareness, and seasonal behavioral patterns, which should be considered when interpreting results. Future research should incorporate clinical data or surveys to validate these trends more robustly.

## Author Contributions

Sitong Li designed the study, conducted data analysis, and drafted the manuscript. Jiacheng Lin and Jiaqi Li assisted with data visualization. Xiaohui Mo and Qiang Ju supervised the study, provided statistical advice, and offered language support.

## Disclosure

The authors have nothing to report.

## Conflicts of Interest

The authors declare no conflicts of interest.

## Data Availability

The data that support the findings of this study are available from the corresponding author upon reasonable request.
